# Methylene Blue has a potent antiviral activity against SARS-CoV-2 and H1N1 influenza virus in the absence of UV-activation in vitro

**DOI:** 10.1038/s41598-021-92481-9

**Published:** 2021-07-12

**Authors:** Valeria Cagno, Chiara Medaglia, Andreas Cerny, Thomas Cerny, Arnaud Charles-Antoine Zwygart, Erich Cerny, Caroline Tapparel

**Affiliations:** 1 Department of Microbiology and Molecular Medicine, University of Geneva, Geneva, Switzerland; 2 Institute of Microbiology, Lausanne University Hospital, University of Lausanne, Lausanne, Switzerland; 3 Epatocentro Ticino, Lugano, Switzerland; 4 Kantonsspital St.Gallen, St.Gallen, Switzerland; 5 Omni Drugs SA, 13 Cours des Bastions, Geneva, Switzerland

**Keywords:** Viral infection, Antiviral agents

## Abstract

Methylene blue is an FDA (Food and Drug Administration) and EMA (European Medicines Agency) approved drug with an excellent safety profile. It displays broad-spectrum virucidal activity in the presence of UV light and has been shown to be effective in inactivating various viruses in blood products prior to transfusions. In addition, its use has been validated for methemoglobinemia and malaria treatment. In this study, we first evaluated the virucidal activity of methylene blue against influenza virus H1N1 upon different incubation times and in the presence or absence of light activation, and then against SARS-CoV-2. We further assessed the therapeutic activity of methylene blue by administering it to cells previously infected with SARS-CoV-2. Finally, we examined the effect of co-administration of the drug together with immune serum. Our findings reveal that methylene blue displays virucidal preventive or therapeutic activity against influenza virus H1N1 and SARS-CoV-2 at low micromolar concentrations and in the absence of UV-activation. We also confirm that MB antiviral activity is based on several mechanisms of action as the extent of genomic RNA degradation is higher in presence of light and after long exposure. Our work supports the interest of testing methylene blue in clinical studies to confirm a preventive and/or therapeutic efficacy against both influenza virus H1N1 and SARS-CoV-2 infections.

## Introduction

Viral pandemics cause significant morbidity and mortality. Influenza A viruses and coronaviruses are among the major human threats due to the presence of an important animal reservoir and the ability of both viruses to cross the species barrier via mutation and reassortment or recombination. The ongoing SARS-CoV-2 (Severe Acute Respiratory Syndrome Coronavirus 2) pandemic unveiled the inability to develop specific antivirals and vaccines in a sufficiently short time frame. Although a limited circulation of influenza virus in the 2020–2021 season was achieved through social distancing and lock-down measures, in the coming years co-infection of SARS-CoV-2 and influenza might contribute to the severity of SARS-CoV-2 pneumonia^1^. Moreover, neuraminidase treatment has been shown to increase the affinity of SARS-CoV-2 for its receptor, ACE-2, and this might increase the susceptibility of influenza infected patients to SARS-CoV-2 superinfection^2^. This scenario highlights the urgent clinical need for broad-spectrum antiviral drugs ready to use during pandemics and epidemics.

Methylene Blue (MB) is an FDA and EMA approved drug with an excellent safety profile. Due to its antimicrobial, anti-inflammatory and antitoxic effects, photoactivated MB is used for a wide range of applications including treatment of methemoglobinemia or malaria^3^. Photoactivated MB is also widely used to obtain blood product preparations free of viruses such as HIV^4^, Ebola, Middle East Respiratory Syndrome coronavirus (MERS)^5^, or SARS-CoV-2 virus^6^. The antiviral activity appears to rely on multiple mechanisms, and is more potent for enveloped viruses^7^. Among other actions, MB is known to corrupt viral DNA or RNA integrity. This is due to a redox reaction in which the molecule accepts electrons on its aromatic thiazine ring, thus being reduced to leuko-methylene blue (MBH_2_) which in turn transfers electrons to other molecules such as nucleic acids. Moreover, MB in combination with oxygen and a source of energy results in the production of singlet oxygen, a highly reactive reaction partner which induces guanine oxidation [8-oxo-7,8-dihydroguanine (8-oxoGua) lesions] damaging DNA or RNA. Other mechanisms include but are not limited to (a) modified carbonyl moieties on proteins, (b) single-strand breaks in the RNA genome and (c) RNA–protein crosslinks, all lesions correlating well with a broad-spectrum virucidal activity^8^. In addition to nucleic acid damaging activity, MB was shown to inactivate viruses such as HIV by targeting also the viral envelope and core proteins^9^.

MB presents few side effects at doses below 5 mg/kg. Long term, high dose administrations such as for malaria treatment, may result in temporary fully reversible blueish coloration of urine, sclera and skin. Sensitivity to thiazine dyes and glucose 6 phosphate dehydrogenase deficiency are contraindications^10^.

Using a classical virus inhibition assay for both H1N1 influenza virus and SARS-CoV-2, we present in vitro data demonstrating a potent antiviral activity of MB with and without UV-activation. We report a strong antiviral activity without light (experiments performed in a closed stainless-steel box) at 2, 4 and 20 h of incubation with the virus. Interestingly, degradation of genomic RNA was mostly observed in presence of light and upon long exposure, confirming that the antiviral effect relies on multiple mechanisms of action and can be efficient also in absence of extensive RNA degradation. Additional experiments carried on with SARS-CoV-2 further highlight that MB is effective also when added on cells already infected. Finally, we demonstrate an additive effect of MB when co-administered with immune sera. Altogether these data open the possibility to evaluate MB as a potential therapeutic agent against influenza virus or SARS-CoV-2 in clinical studies.

## Results

In order to define a non-toxic dose range of MB, we calculated the 50% cytotoxic concentration (CC_50_) in our in vitro infection systems, MDCK for H1N1 and Vero-E6 cells for SARS-CoV-2. We assessed MB toxicity in infection medium and upon different times of administration, according to the experimental conditions chosen to test the antiviral activity of the compound (Table1).

**Table 1:** Assessment of MB toxicity.

Cell line	Infection medium	Hours of treatment	CC50 (µg/ml)
MDCK	Serum-free DMEM + 0.2 µg/ml TPCK trypsin	1*	46.44
		48	23.22
Vero-E6	DMEM + 5% FCS	1*	84.78
		48	46.14

*Cell viability was measured 48 h after treatment.

**Figure 1:**
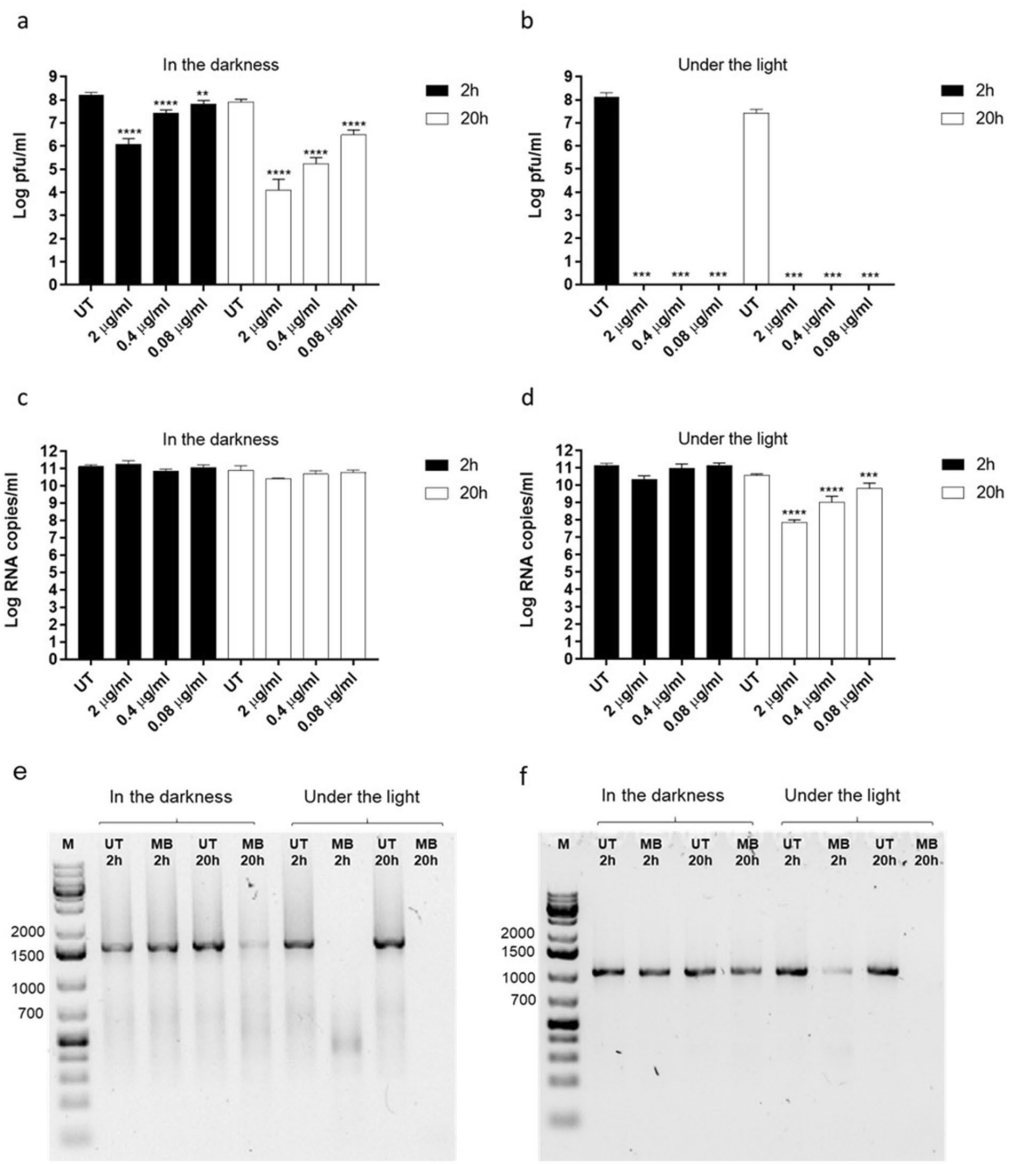
Assessment of MB antiviral activity on human H1N1. MB was incubated with H1N1 virus A/Netherlands/602/2009 (1.5 × 10^7^ pfu) for 2 h or 20 h in the darkness (**a, c**) or in the presence of light (**b**, **d**). At the end of the incubation, mixtures were serially diluted and added for 1 h at 37 °C on MDCK cells (**a**) and (**b**). Mixtures were then removed and the cells were overlaid with medium containing 0.8% agarose and TPCK trypsin 1 µg/ml. At 48 h post infection (hpi) the cells were fixed in order to count the plaques and determine the viral titer in each experimental condition. In parallel, viral RNA was quantified by RT-qPCR (**c, d**) and viral genome integrity was assessed by PCR amplification of the NP (**e**) and M (**f**) viral segments. In (**e**) and (f) only the “UT” and “MB 2 µg/ml” conditions were examined. Results are mean and SD of two independent experiments. 2 µg/ml (6.25 µM), 0.4 µg/ml (1.25 µM), 0.08 µg/ml (0.25 µM). UT = untreated. **p < 0.01, ***p < 0.001, ****p < 0.0001.

We first asked whether MB was able to exert antiviral activity independently of visible light. We thus incubated different doses of the compound spanning from 2 μg/ml to 0.08 μg/ml, with 1.5 × 10^7^ plaque forming units (pfu) of human A/Netherlands/602/2009 (H1N1), for 2 h or 20 h at room temperature and in the presence or absence of visible light. We then measured the number of infectious particles in each condition. MB exerted virucidal activity in the presence and absence of light and at both incubation times, suggesting that the compound can irreversibly inactivate a relevant enveloped respiratory virus independently of visible light (Fig. 1a,b). As more than one mechanism of action can underlie the antiviral properties of MB^6^, we asked whether the compound would damage the H1N1 genome. We measured genome integrity by RT-qPCR in each of the above-described conditions (Fig. 1c,d). Surprisingly, the reduction of H1N1 infectiousness induced by MB did not always result in a reduction of viral genome quantified by RT-qPCR. Only upon 20 h incubation under the light and at the highest doses, MB induced a significant decrease in viral RNA, compared to the untreated control (Fig. 1d). However, as the RT-qPCR amplicon is only ~ 105 bp, it could remain intact while longer genomic fragments may be affected by the treatment. We therefore investigated the integrity of H1N1 segments by conventional PCR, targeting the entire NP and M genes, which are 1.5 and 0.9 Kb long respectively (Fig. 1e,f). As expected, the highest tested dose of MB (2 µg/ml), caused significant degradation of these two viral segments after 20 h incubation in the light. Interestingly, partial degradation was also observed upon 20 h incubation in the darkness and upon 2 h incubation under the light, while no degradation was observed after 2 h incubation in the dark (Fig. 1e,f). These findings suggest that RNA damage caused by MB^6,7^ does not fully account for its antiviral activity. Overall these preliminary results prompted us to evaluate the virucidal activity of MB against SARS-CoV-2.

**Figure 2:**
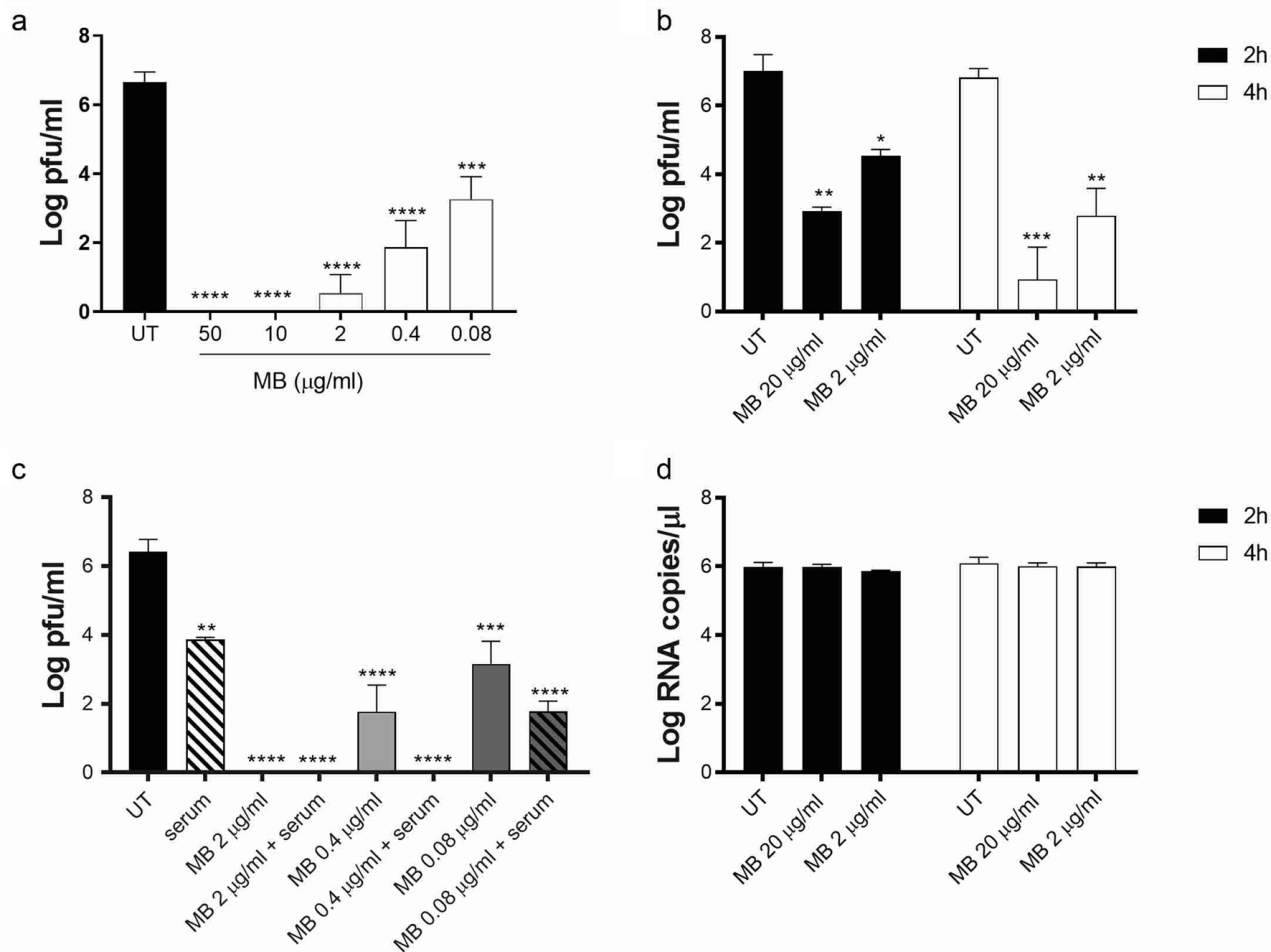
Assessment of MB antiviral activity on SARS-CoV-2. MB was incubated with SARS-CoV-2 (5 × 10^6^ pfu) for 20 h (**a**,**c**) or 2 h and 4 h (**b**, **d**) in the darkness, in (**c**) MB was incubated in combination with human convalescent serum (diluted 1:80). At the end of the incubation, mixtures were serially diluted and added for 1 h at 37 °C on Vero-E6 cells. Mixtures were then removed and cells were overlaid with medium containing 0.8% avicel. Cells were fixed 48 hpi and plaques were counted in order to determine the viral titer in presence or absence of MB. In parallel, viral RNA was quantified by RT-qPCR (**d**). Results are mean and SD of three independent experiments. 50 µg/ml (156 µM), 10 µg/ml (31.2 µM), 2 µg/ml (6.25 µM), 0.4 µg/ml (1.25 µM), 0.08 µg/ml (0.25 µM). UT = untreated. *p < 0.05, **p < 0.01 ***p < 0.001 ****p < 0.0001.

Hence, we incubated different doses of the drug, ranging between 50 μg/ml and 0.08 μg/ml, with 5 × 10^6^ pfu of SARS-CoV-2 for 20 h in the dark and at room temperature. The results shown in Fig. 2 evidence a complete inhibition of SARS-CoV-2 replication from 50 to 10 µg/ml and a 3.05 log reduction at 0.08 µg/ml, the lowest dose tested (Fig. 2a). Of note Jin et al.^6^ observed no virucidal activity of MB against SARS-CoV-2 when administered for 40 min in the dark. We thus performed additional experiments incubating MB and the virus for 2 h or 4 h and evidenced a statistically significant effect at the 2 h incubation time (Fig. 2b). This effect was however lower compared to that observed at 20 h incubation (5.3 log reduction after 2 h treatment with 20 µg/ml and 3.6 log with 2 µg/ml).

Subsequently we evaluated the possible combinatorial activity of MB and immunoglobulins present in the serum of a convalescent individual. Preliminary tests were conducted to identify the optimal serum dilution able to neutralize the virus and evidenced a 4.15 log decrease in presence of 1:16 dilution, a 2.3 log decrease in presence of 1:80 dilution, and absence of neutralization at lower serum dilutions. In order to be able to evaluate any additive effect, the serum was diluted 1:80 for further experiments. 5 × 10^6^ pfu of SARS-CoV-2 were incubated for 20 h in the dark with three different doses of MB in presence or absence of diluted convalescent serum. The results (Fig. 2c) evidence a partial additive effect of MB and convalescent sera, with a complete inhibition of viral infectivity when combining MB (up to 0.4 µg/ml) and serum. To rule out the possibility that serum factors other than anti-SARS-CoV-2 antibodies could enhance the action of MB, we also tested MB in combination with a non-immune control serum, which showed no anti-SARS-CoV-2 activity alone nor potentiation of MB effect (Supplementary Fig. S1). To further verify the mechanism of action of MB in the dark against SARS-CoV-2, we performed RT-qPCR on the samples incubated for 2 h and 4 h. Despite the logarithmic decrease in viral infectivity (Fig. 2b), no decrease of viral RNA was evidenced by RT-qPCR (Fig. 2d).

**Figure 3:**
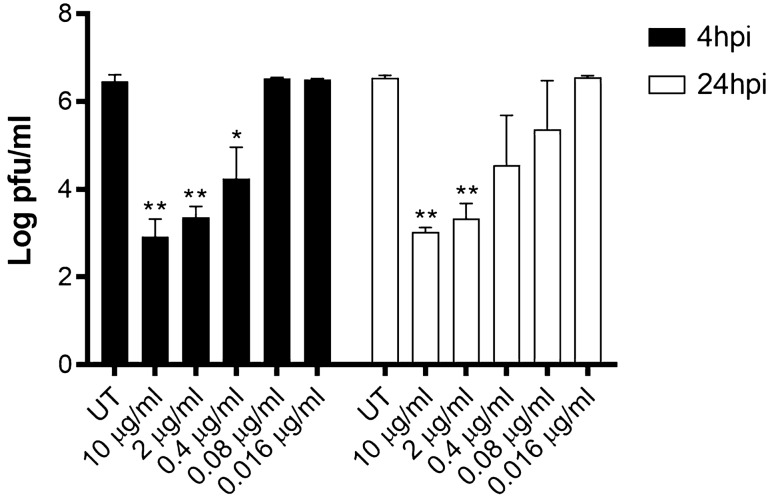
Viral yield reduction assay. Vero-E6 were infected with SARS-CoV-2 (MOI 0.005). 4 h or 24 hpi, medium containing or not serial dilutions of MB was added on cells. 24 h later, cell supernatants were collected and titrated on Vero-E6 cells as described in Fig. 2. Results are mean and SD of two independent experiments performed in duplicate. *p < 0.05, **p < 0.01.

Finally, we assessed the efficacy of MB for therapeutic use. We thus tested whether MB was able to reduce the viral yield in cells previously infected with SARS-CoV-2. The treatment was administered at 4 or 24 hpi and the virus released by the infected cells was measured titrating the culture supernatants at 48 hpi. In both conditions we observed viral inhibition in a dose–response manner (Fig. 3) with an EC_50_ of 0.11 μg/ml at 4 hpi and of 0.13 μg/ml at 24 hpi.

## Discussion

Here we show broad-spectrum virucidal activity of MB in absence of light activation, against both H1N1 influenza virus and SARS-CoV-2 after incubation times of 2, 4 or 20 h. In previous studies^6^, no antiviral activity was observed after shorter incubations (40 min) in absence of light. However, in these works, the evaluation of viral titer was done with qPCR and not by measuring infectivity. The activity of the compound in absence of UV-activation is thus suggestive of a different mechanism of action than that reported in presence of UV light or slower kinetics. This hypothesis is further sustained by the lack of H1N1 NP or M RNA decrease and the detection of both H1N1 and SARS-CoV-2 by RT-qPCR after a 2 h incubation with MB in the darkness (Figs. 1d and 2d), which is in line with previous reports. We highlight that the antiviral activity of MB is enhanced in combination with convalescent serum, and that such effect is specifically due to the presence of immunoglobulins against Sars-Cov-2. This suggests that the compound could not only be effective in infected patients, but also that its efficacy could be further increased by immune serum.

A limitation of our study with SARS-CoV-2 is that the experiments are performed in Vero-E6 cell line where other drugs, such as hydroxychloroquine^11^, were reported to exert antiviral activity, however without showing efficacy in clinical trials^12^. Nevertheless, in those cases the drug interfered with viral replication inside the cell, while MB acts also on extracellular viral particles. Therefore, we do not expect to observe such a discrepancy when going into clinical trials.

A recent French publication on a cohort of 2500 end stage cancer patients treated with MB during the first wave of CoviD-19 mentions a possible protective role of MB against respiratory viruses, as in this cohort there were no reported cases of influenza or SARS-CoV-2 infections^13^. Another report describes a possible complementary effect of MB in vitro on the SARS-CoV-2 Spike–ACE2 protein–protein interaction^14^. The proposed mechanism of action is mediated by the binding of MB on the cells and not on the virus. The experiments were performed in a different experimental setting than ours, by pre-treating the cells with MB and subsequently infecting them with SARS-CoV-2. Lastly, an additional study compared in vitro the median Effective Concentration (EC50) of 3 drugs against SARS-COV-2, when administered 4 h before infection. MB was the most efficient antiviral drug with an EC50 of 0.30 ± 0.03 µM^15^. This study did not focus on the activity in absence or presence of light, nor in measuring the viral inactivation. The drug was added 4 h before infection and left on cells for the entire experiment, and the infectivity was evaluated through RT-qPCR. The EC50 determined was however in line with our experiments in which we added MB after infection (Fig. 3), validating our results.

In view of the wide variety of singlet oxygen driven chemical reactions corroding nucleic acids and proteins, one is surprised by the well documented absence of severe side effects of MB, even at doses up to 5 mg/kg. This may be linked to the fact, that the body’s immune system uses MB for its own purposes. It is known that antibodies have, close to their binding site, a catalytic site capable of producing singlet oxygen in the presence of water^16^. Riboflavin, also known as Vitamin B2 is another efficient singlet oxygen producing compound^17^. It seems reasonable to assume that mammals have developed so far unknown control mechanisms to keep singlet oxygen collateral damages at bay.

Altogether, our results support the interest of testing MB in clinical studies to confirm a preventive or therapeutic efficacy, alone or combined with immune sera, against two major public health concerns: influenza virus and SARS-CoV-2^1^.Further experiments will be required to fully understand the different antiviral mechanisms of action of MB against these two enveloped viruses. Based on previously published data, MB might inactivate viruses limiting viral replication and spread beyond the upper respiratory tract. In addition, its anti-inflammatory activity^3^ may lower the side effects linked to the host response.

## Materials and methods

### Compound and convalescent serum

Methylene blue (Methylthonium chloride solution) was purchased from ProVepharm. A convalescent or a control serum, respectively tested positive or negatve for IgG against SARS-CoV-2 by ELISA, were isolated from blood collected in serum separator tubes. Within 1 h from collection, sera were isolated by centrifuging the blood at 2500 RPM for 15 min. The sera were aliquoted and stored at − 80 °C.

### Cells and virus

MDCK and (ATCC CCL34) cells and Vero C1008 (clone E6) (ATCC CRL-1586) cells (kindly provided by Prof Gary Kobinger from the University of Laval), were propagated in DMEM High Glucose + Glutamax supplemented with 10% fetal bovine serum (FBS) and 1% penicillin/streptavidin (pen/strep).

SARS-CoV-2/Switzerland/GE9586/2020 was isolated from a clinical specimen in the University Hospital of Geneva in Vero-E6. Cells were infected and supernatant was collected 3 days post infection, clarified, aliquoted and frozen at − 80 °C before titration by plaque assay in Vero-E6.

Human H1N1 virus A/Netherlands/602/2009, clade 1A.3.3.2, [Fludb entry CY148018]^18^ was a gift from Prof. Mirco Schmolke (University of Geneva) and was cultivated and titrated in MDCK cells by plaque assay.

### Toxicity assay

Vero-E6 and MDCK cells (13,000 and 20,000 cells per well respectively) were seeded in 96-well plate one day before the assay. For toxicity evaluation in Vero-E6 methylene blue was serially diluted 1:2 in DMEM supplemented with 5% FBS and added on cells for 1 h, followed by a washout and the addition of DMEM supplemented with 5% FBS for additional 48 h hours. Alternatively, the dose range of MB was added on the cells for 48 h. The toxicity evaluation in MDCK cells was performed in serum free DMEM supplemented with 0.2 µg/ml of TPCK-Trypsin (Sigma), using the same MB dose-range and times of treatment applied to Vero-E6. MTS reagent (Promega) was added on cells for 3 h at 37 °C according to manufacturer instructions, subsequently absorbance read at 570 nm. Percentages of viability were calculated by comparing the absorbance in treated and untreated wells. 50% cytotoxic concentration (CC_50_) were calculated with Prism 8 (GraphPad).

### Plaque assay

Vero-E6, 100000 cells per well, were seeded in 24-well plate one day before the assay. Serial dilutions of SARS-CoV-2 were added on cells in a final volume of 200 µl for 1 h at 37 °C. After infection Vero-E6 cells were washed and overlaid with 0.8% Avicel rc581 in medium supplemented with 5% FBS.

MDCK cells 500000 cells per well, were seeded in 6-well plate 24 h in advance. Serial dilutions were added on cells in a final volume of 500 µl for 1 h at 37 °C. MDCK monolayers were then washed and overlaid with 0.8% agarose in medium supplemented with TPCK trypsin 1 µg/ml.

Two days after infection, cells were fixed with paraformaldehyde 4% and stained with crystal violet solution containing ethanol.

Plaques were counted.

### Inhibition assay

H1N1 (1.5 × 10^7^ pfu) or SARS-CoV-2 (5 × 10^6^ pfu) were incubated at room temperature in the dark (in an opaque stainless-steel box) or under visible light, in a final volume of 100 µl, with different dilutions of MB for 2 h or 20 h. The infection mixtures were then serially diluted (1:10 factor) and added for 1 h at 37 °C on the respective host cell lines. The residual infectivity was evaluated by plaque assay as described in the previous paragraph. The residual infectivity of the different conditions was calculated. Statistical analysis was done with Prism software (GraphPad) comparing the different conditions.

### Neutralization assay

SARS-CoV-2 (5 × 10^6^ pfu) was incubated at room temperature in the dark for 20 h in a final volume of 400 µl, with different dilutions of immune serum. The infection mixtures were then serially diluted (1:10 factor) and added for 1 h at 37 °C on the respective host cell lines and subjected to plaque assay as described in the paragraph above. The same experiments were conducted in presence of immune serum and non immune serum at dilution 1:80 in presence or absence of different concentrations of MB.

### RT-qPCR analysis

H1N1 (1.5 × 10^7^ pfu) was incubated at room temperature in the dark (in an opaque stainless steel box) or under visible light, in a final volume of 100 µl, with different dilutions of MB for 2 h or 20 h. SARS-CoV-2 (5 × 10^6^ pfu) was incubated in the dark with 2 or 20 μg/ml of MB for 2 or 4 h. Viral RNA was extracted with EZNA viral extraction kit (Omega Biotek) and quantified by using RT-qPCR (Table 2) with the QuantiTect kit (Qiagen) in a StepOne ABI Thermocycler.

**Table 2:** Primers and probes used for RT-qPCR analysis of A(H1N1)pdm09 virus and SARS-CoV-2.

Target	Primer or probe	Sequence (Taqman at 60 °C)	Amplicon length
H1N1 M gene	InfA-CDC-For	GACCRATCCTGTCACCTCTGAC	105 bp
	InfA-CDC-For	AGGGCATTYTGGACAAAKCGTCTA	
	InfA-Probe	FAM-TGCAGTCCTCGCTCACTGGGCACG-BHQ1	
Sars-CoV-2 E gene	SarsCov-2-For	ACAGGTACGTTAATAGTTAATAGCGT	112 bp
	SarsCov-2-Rev	ATATTGCAGCAGTACGCACACA	
	SarsCov-2-Probe	FAM-ACACTAGCCATCCTTACTGCGCTTCG-BBQ	

### PCR analysis

Viral RNAs were isolated from H1N1 (1.5 × 10^7^ pfu) incubated at room temperature in the dark (in an opaque stainless-steel box) or under visible light, in a final volume of 100 µl, with MB (2 µg/ml) for 2 h or 20 h. Samples were then reverse transcribed. The RT reaction was performed with a mix composed of First Strand Buffer 5x (Invitrogen), H2O Rnase free, Superscript ® III RT/Platinum ® Taq Mix (Invitrogen), 0.1 M DTT (Invitrogen), dNTPs (25 mM), Protector RNase inhibitor (40U/ul) (Roche) and Random hexamers (50ug/ul) (Invitrogen).

Viral gene segments were then amplified from the viral cDNA using a master mix composed of 10X PCR Rxn buffer (-MgCl2) (Invitrogen), 50 mM MgCl_2_ (Invitrogen), H_2_O Rnase free, Platinum Taq DNA polymerase (5U/ul) (Invitrogen), dNTPs (10 mM) and M13-tailed primers specific for the M and NP H1N1segments (Table 3). The length and the quality of the amplified fragments were verified by electrophoresis in an agarose gel 1% with 4 ul of Sybr-safe.

**Table 3:** Primers used to amplify gene segments of A(H1N1)pdm09 virus.

Gene	Primer	Binding position	Sequence*
M	Forward	1–21	**TGTAAAACGACGGCCAGT**ATGAGTCTTCTAACCGAGGTC
M	Reverse	959–982	**CAGGAAACAGCTATGACC**TTACTCTAGCTCTATGTTGACAAA
NP	Forward	1–20	**TGTAAAACGACGGCCAGT**ATGGCGTCTCAAGGCACC
NP	Reverse	1474–1494	**CAGGAAACAGCTATGACC**TCAACTGTCATACTCCTCTGC

*The sequence of the M13 tail is indicated in bold.

### Viral yield reduction assay

Vero-E6 cells (100,000 cells per well) were seeded in 24-well plate. Cells were infected with SARS-CoV-2 (MOI, 0.005 pfu/cell) for 4 h at 37 °C. The monolayers were then washed and overlaid with medium supplemented with 5% FBS containing serial dilutions of compound, alternatively the compound was added 24 hpi. Supernatants were harvested 48 hpi and titrated on Vero-E6 cells.

### Statistical analysis

Where possible, half-maximal antiviral effective concentration (EC50) values were calculated by regression analysis using the dose–response curves generated from the experimental data using Prism software (GraphPad). The 50% cytotoxic concentration (CC_50_) was determined using logarithmic viability curves. Two-way or one-way ANOVA, followed by Dunnet’s multiple comparison test, was used to assess the statistical significance of the differences between treated and untreated samples. Significance was set at the 95% level.

### Ethical authorization

Written informed consent was obtained from the subjects involved and the research protocol was approved by the local ethics committee (commission cantonale d’éthique de la recherche CCER de Genève) and performed in accordance with relevant regulations and with the Declaration of Helsinki.

## Supplementary Information

Supplementary Information.

## Data Availability

Raw data are available upon request to the authors.
