# Supplementary material for: Methylene Blue has a potent antiviral activity against SARS-CoV-2 and H1N1 influenza virus in the absence of UV-activation in vitro
Source: Sci Rep. 2021 Jul 12;11:14295. doi: 10.1038/s41598-021-92481-9 (PMC8275569; doi:10.1038/s41598-021-92481-9)
Supplement: Supplementary file 1 — Supplementary Information. [file 41598_2021_92481_MOESM1_ESM.pdf]

Supplementary information

Methylene Blue has a potent antiviral activity against SARS-CoV-2 and H1N1 influenza virus in the absence of UV-activation in vitro

Short title: Methylene Blue antiviral activity against SARS-CoV-2 and influenza A H1N1

Valeria Cagno[1°,2], Chiara Medaglia[1°], Andreas Cerny[3], Thomas Cerny[4], Arnaud Charles-Antoine Zwygart[1], Erich Cerny[#*5], Caroline Tapparel[#*1],

[1] Department of Microbiology and Molecular Medicine, University of Geneva, Geneva, Switzerland

[2] Institute of Microbiology, Lausanne University Hospital, University of Lausanne, Lausanne, Switzerland

[3] Epatocentro Ticino, Lugano, Switzerland

[4] Kantonsspital St.Gallen, St.Gallen, Switzerland,

[5] Omni Drugs SA, 13 Cours des Bastions, Geneva, Switzerland

* correspondence to : Caroline Tapparel (caroline.tapparel@unige.ch), Erich Cerny (eh.cerny@gmail.com)

°, [#] these authors contributed equally to this work

SUPPLEMENTARY FIGURE

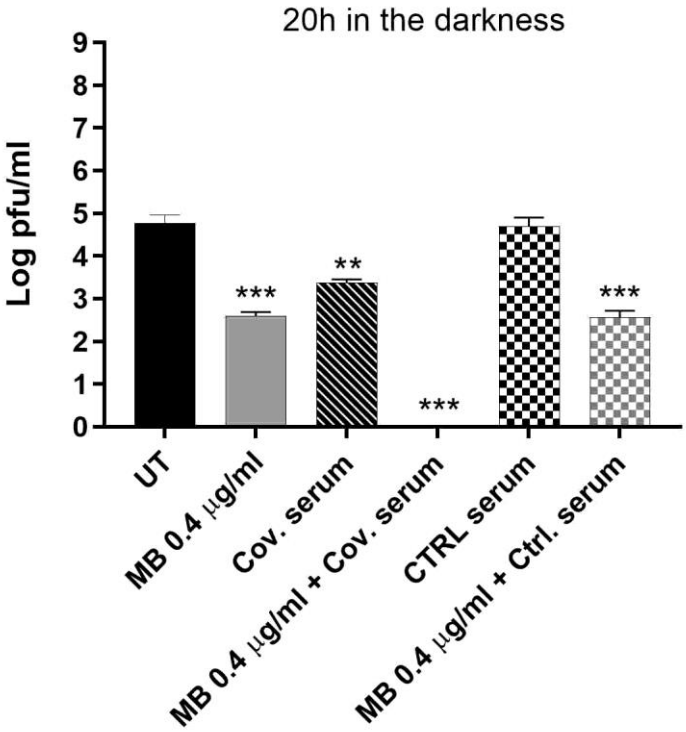

Figure S1. **Assessment of the additive effect of MB and convalescent sera effect against SARS-CoV-2.** SARS-CoV-2 (1*105 pfu) was incubated for 20h in the darkness, with MB (0.4 μg/ml), with human convalescent serum (Cov. Serum, diluted 1:80), with MB + Cov. Serum, with control serum negative for Sars-Cov2 Ab (CTRL serum, diluted 1:80), with MB + CTRL serum, or in absence of treatments (UT). At the end of the incubation, mixtures were serially diluted and added for 1h at 37°C on Vero-E6 cells. Mixtures were then removed and cells were overlaid with medium containing 0.8% avicel. Cells were fixed 48 hpi and plaques were counted in order to determine the viral titer in presence or absence of MB. Results are mean and SD of two independent experiments performed in duplicate. **p<0.01 ***p<0.001.
